# Development of the CDISC Pediatrics User Guide: a CDISC and conect4children collaboration

**DOI:** 10.3389/fmed.2024.1370916

**Published:** 2024-06-17

**Authors:** John Owen, Anando Sen, Beate Aurich, Corinna Engel, Giacomo Cavallaro, Eva Degraeuwe, Dipak Kalra, Ronald Cornet, Melissa Walsh, Teresa Berkery, Avril Palmeri, Fenna Mahler, Salma Malik, Laura Persijn, Chima Amadi, Jacques Thuet, Simon Woodworth, Sinead Nally, Rebecca Leary, Richard Marshall, Volker Straub

**Affiliations:** ^1^CDISC Europe Foundation, Brussels, Belgium; ^2^John Walton Muscular Dystrophy Research Centre, Translational and Clinical Research Institute, Newcastle University and Newcastle Hospitals NHS Foundation Trust, Newcastle upon Tyne, United Kingdom; ^3^Drug Safety Consulting S.A.S.U., Paris, France; ^4^Center for Pediatric Clinical Studies, University Children’s Hospital Tübingen, Tuebingen, Germany; ^5^NICU, Fondazione IRCCS Ca’ Granda Ospedale Maggiore Policlinico, Milan, Italy; ^6^Departement of Internal Medicine and Pediatrics, Ghent University, Ghent, Belgium; ^7^Ghent University Hospital (UZ Gent), Ghent, Belgium; ^8^The European Institute for Innovation through Health Data, Ghent, Belgium; ^9^Department of Medical Informatics, Amsterdam University Medical Centers, University of Amsterdam, Amsterdam, Netherlands; ^10^Amsterdam Public Health, Digital Health and Methodology, Amsterdam, Netherlands; ^11^INFANT Research Centre, University College Cork, Cork, Ireland; ^12^Department of Pharmacy, Division Pharmacology and Toxicology, Radboud University Medical Center, Nijmegen, Netherlands; ^13^The European Clinical Research Infrastructure Network, Paris, France; ^14^Belgian Paediatric Clinical Research Network (BPCRN), Ghent University, Ghent, Belgium; ^15^Institut de Recherches Internationales Servier, Suresnes, France; ^16^Novartis Pharmaceuticals, Dublin, Ireland; ^17^Accurate Systems Limited, Saffron Walden, United Kingdom

**Keywords:** CDISC, data standards, data dictionary, paediatric clinical trials, interoperability

## Abstract

**Introduction:**

The conect4children (c4c) project aims to facilitate efficient planning and delivery of paediatric clinical trials. One objective of c4c is data standardization and reuse. Interoperability and reusability of paediatric clinical trial data is challenging due to a lack of standardization. The Clinical Data Interchange Standards Consortium (CDISC) standards that are required or recommended for regulatory submissions in several countries lack paediatric specificity with limited awareness within academic institutions. To address this, c4c and CDISC collaborated to develop the Pediatrics User Guide (PUG) consisting of cross-cutting data items that are routinely collected in paediatric clinical trials, factoring in all paediatric age ranges.

**Methods and Results:**

The development of the PUG consisted of six stages. During the scoping phase, subtopics (each containing several clinically relevant concepts) were suggested and debated for inclusion in the PUG. Ninety concepts were selected for the modelling phase. Concept maps describing the Research Topic and representation procedure were developed for the 19 concepts that had no (or partial) previous modelling in CDISC. Next, metadata and implementation examples were developed for concepts. This was followed by a CDISC internal review and a public review. For both these review stages, the feedback comments were either implemented or rejected based on budget, timelines, expert review, and scope. The PUG was published on the CDISC website on February 23, 2023.

**Discussion:**

The PUG is a first step in bridging the lack of child specific CDISC standards, particularly within academia. Several academic and industrial partners were involved in the development of the PUG, and c4c has undertaken multiple steps to publicize the PUG within its academic partner organizations – in particular, the European Reference Networks (ERNs) that are developing registries and dictionaries in 24 disease areas. In the long term, continued use of the PUG in paediatric clinical trials will enable the pooling of data from multiple trials, which is particularly important for medical domains with small populations.

## 1 Introduction

Paediatric clinical trials face significant challenges, including ethical issues, (very vulnerable populations, such as neonates, too young to decide for their own, etc.) age-specific dosages, palatable medications, and specific populations (e.g., neonates, toddlers, children, adolescents) (1–3). Multi-national trials are additionally impacted by site-specific considerations and differences in consent rules (4, 5). The European Union (EU) and Innovative Medicines Initiative (IMI) funded conect4children (c4c) project was initiated in 2018 to facilitate efficient planning and delivery of paediatric clinical trials (6). A working group within c4c deals with the standardization and harmonization of paediatric clinical trial data. A major focus of this working group has been to foster collaborations among large initiatives, academic and industry partners and to enable data interoperability (7).

c4c engaged with the Clinical Data Interchange Standards Consortium (CDISC) as a third party to develop paediatric data standards. CDISC is an organization founded in 1997 to develop standards to link clinical research data to healthcare data and, through that process, harmonize the transfer of clinical research data for analysis and regulatory review. CDISC standards are required for regulatory submissions in the United States and Japan, while they are recommended in the European Union and China (8–11). This ensures that any new standard, dictionary, or tool that is developed with CDISC will be immediately available for regulatory submissions in a large part of the world.

Due to the lack of paediatric specificity of CDISC standards, it is difficult for any organization to fully adopt CDISC standards when conducting paediatric clinical studies (12–14). This leads to institutions developing proprietary in-house data standards that cannot be shared outside the institutions and, in turn, prevents interoperability of acquired data. In addition, there is limited awareness of CDISC standards within academia (15). An internal survey with c4c academic partners in 2019 showed that only 32% of the institutions used CDISC standards, while 44% had never heard about CDISC. One of the goals of c4c has been to familiarize academia with CDISC standards. To tackle these challenges, a decision was taken to develop a CDISC Pediatrics User Guide (PUG) consisting of cross-cutting terms collected routinely in paediatric clinical trials with inputs from c4c academic and industry partners.

CDISC standards include foundational (16) and therapeutic area (TA) standards (17). The foundational standards form the basis of the entire CDISC suite of standards that focus on the core principles for defining data and include models, domains, and specifications for data representation. The TA standards extend the foundational standards to represent data related to specific disease areas. TA standards include disease-specific metadata, guidance, and examples on implementing CDISC standards. Currently, CDISC has 49 TA User Guides (TAUGs).

Two major components of CDISC foundational standards are Clinical Data Acquisition Standards Harmonization (CDASH) and Study Data Tabulation Model (SDTM). The CDASH model defines a standard structure for the organization, naming and description of variables and associated attributes to support data Research Topic in clinical trials. In combination with its associated Implementation Guide (the CDASHIG), it establishes a standard template for collecting data consistently across studies, sponsors, and sites (18). The SDTM provides a standard structure for the tabulation of study data. In combination with its associated Implementation Guide (the SDTMIG), it establishes a standard for organizing and formatting data, which facilitates the processes for the management, analysis and reporting of study data. Implementing SDTM supports data aggregation, enables data sharing and reuse, and improves the regulatory approval process (19, 20). Furthermore, use of data Research Topic formats and structures defined in the CDASH model and the CDASHIG ensures a clear traceability of collected data that is represented in standard tabulation format according to the SDTM and the SDTMIG.

While CDASH and SDTM may represent largely overlapping data, there are subtle differences. For example, missing data in CDASH implies data has not been recorded, while in SDTM, missing data implies its absence has been validated. Validation is performed by asking specific questions as mandated by CDASH. A missing adverse event in CDASH by itself might mean that no adverse events were experienced, or it might mean that adverse events were experienced but not recorded. To validate this, a mandatory specific question is asked: “Were any adverse events experienced?” Based on the answer to this question, absence of adverse events in SDTM indicates that no adverse events were experienced (21).

This paper describes the steps involved in the development of the PUG. While user guides and TAUGs are freely available on the CDISC website, the rigorous methodology to get to the final version has not been explicitly described. The primary objective of this article is to share knowledge with sponsors of paediatric clinical trials, especially those in academia, and to encourage the adoption of paediatric CDISC standards. We wish to achieve this by providing a comprehensive description of the method, enabling them to become familiar with its nuances and aid in successfully implementing CDISC standards.

## 2 Methods

Despite eventually being housed in CDISC’s TAUG webpage, it should be noted that the PUG was named as a “User Guide” and not as a TAUG to reinforce the fact that it is cross-cutting and not related to one specific therapeutic area. The PUG uses CDASH to generate a user-friendly interface for case report forms (CRFs) and SDTM to store validated data in a machine-readable format that can be further used for analysis.

A team consisting of CDISC standards experts, paediatric clinical research experts from CDISC member organizations, and paediatric subject matter experts (SMEs) from the c4c consortium was formed in January 2021 that met virtually 44 times during the development of the PUG. Development of the user guide followed a consensus-based, clinical data standards development process consisting of six stages (22):

1. Scoping – Identification of development topics
2. Concept Modelling – Deep dive understanding of topics
3. Standards Development – Development of standards content and implementation examples
4. Internal Review – Targeted review
5. Public Review – User community review
6. Publication – Freely available on the CDISC website.

Three major conditions were imposed on the selection of data items. Any item selected for the PUG must be (i) cross-cutting (disease agnostic), (ii) specific to paediatrics (or subcategory of paediatrics such as neonates or adolescents), and (iii) collected in clinical trials. The PUG would cover five broad areas:

1. Information about the study subjects (e.g., demographics, vital signs, medical conditions, treatment, reproductive development, diet and nutrition);
2. Information about the study subject’s family (e.g., background, medical conditions, substance use);
3. Pregnancy and birth (e.g., pregnancy and birth events, multiple births);
4. Study conduct (e.g., study identifiers, elements and arms, protocol milestones, visits, eligibility criteria);
5. Links to questionnaires, ratings, and scales (QRS) supplements of interest to paediatric research.

User guides within CDISC are organized by broad topics, as mentioned above. These topics are then expanded to include subtopics and further into concepts. Within subtopic descriptions, examples are provided on how to represent concepts related to that subtopic within CDISC standards. For example, information about the subject is a broad topic, demographic information is a subtopic, and date of birth, phenotypic sex, race, and age are concepts. Both subtopics and concepts can be nested. For example, pubertal status is a subtopic within the subtopic development and has concepts of male genitalia stage, female breast stage, etc. within it. Date of menarche and age at menarche are concepts within the concept of menarche.

### 2.1 Scoping

**Figure 1:**
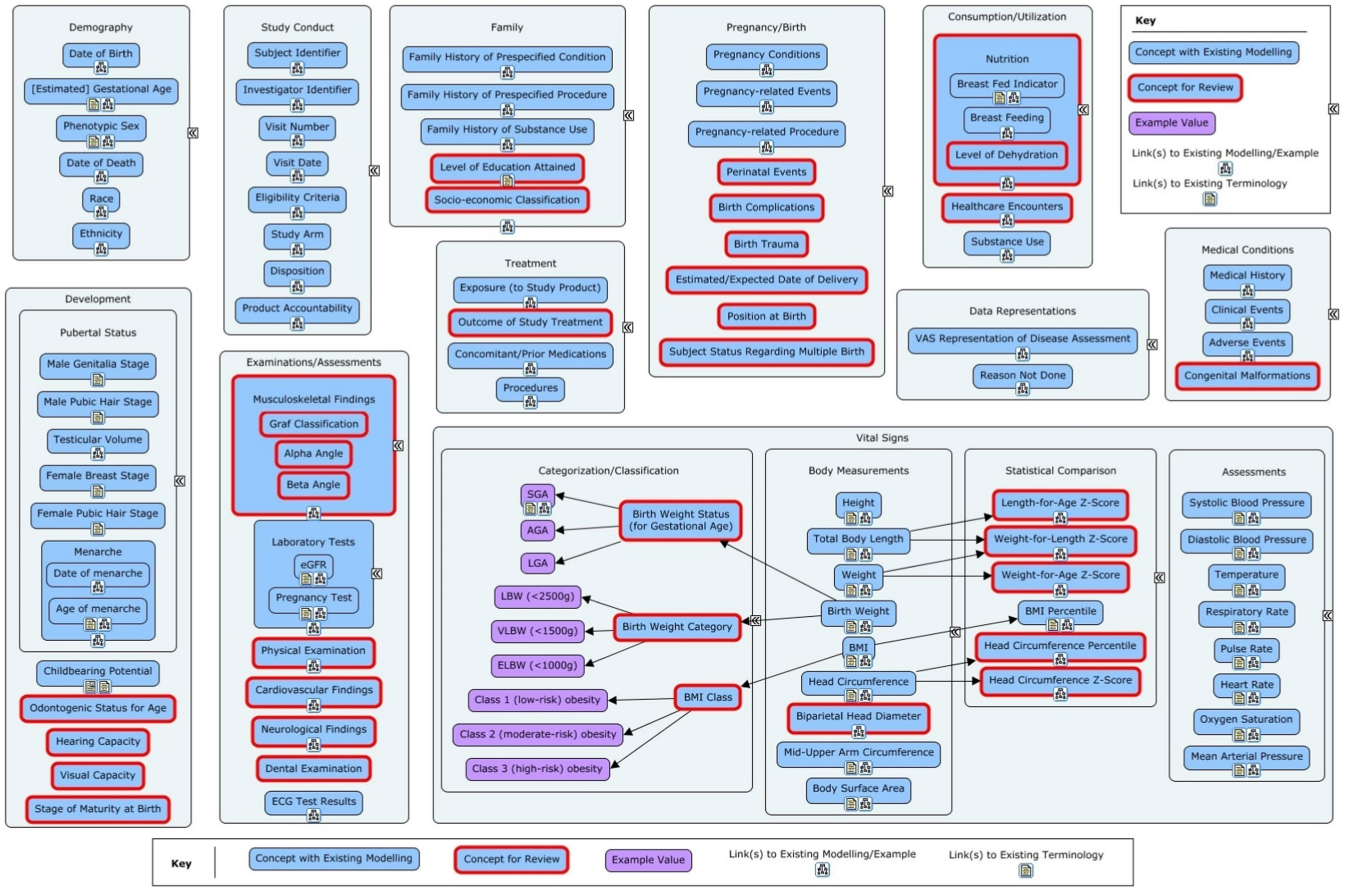
Overview of the non-Questionnaires, Rating and Scales (QRS) concepts in the initial concept list for the Pediatrics User Guide. The red-outlined concepts marked for review had partial or no modelling available or required a deeper dive to fully understand the data for the concept.

The scoping phase lasted between January 2021 and July 2021. Several inputs were used in suggesting concepts and data items for the PUG. These included the c4c cross-cutting paediatric data dictionary (CCPDD) version 1 along with the items that were deferred to version 2 of the CCPDD (23), the National Cancer Institute Enterprise Vocabulary Services (NCI-EVS) paediatric terminology (24), data items collected from the c4c proof of viability studies (25), and expert opinions provided by c4c partners (Bayer, Ghent University, and Drug Safety Consulting S.A.S.U., Paris). There were 135 concepts at this stage. Visual representations of these concepts within their respective subtopics are shown in Figure 1 (non-QRS concepts) and 2 (QRS concepts).

**Figure 2:**
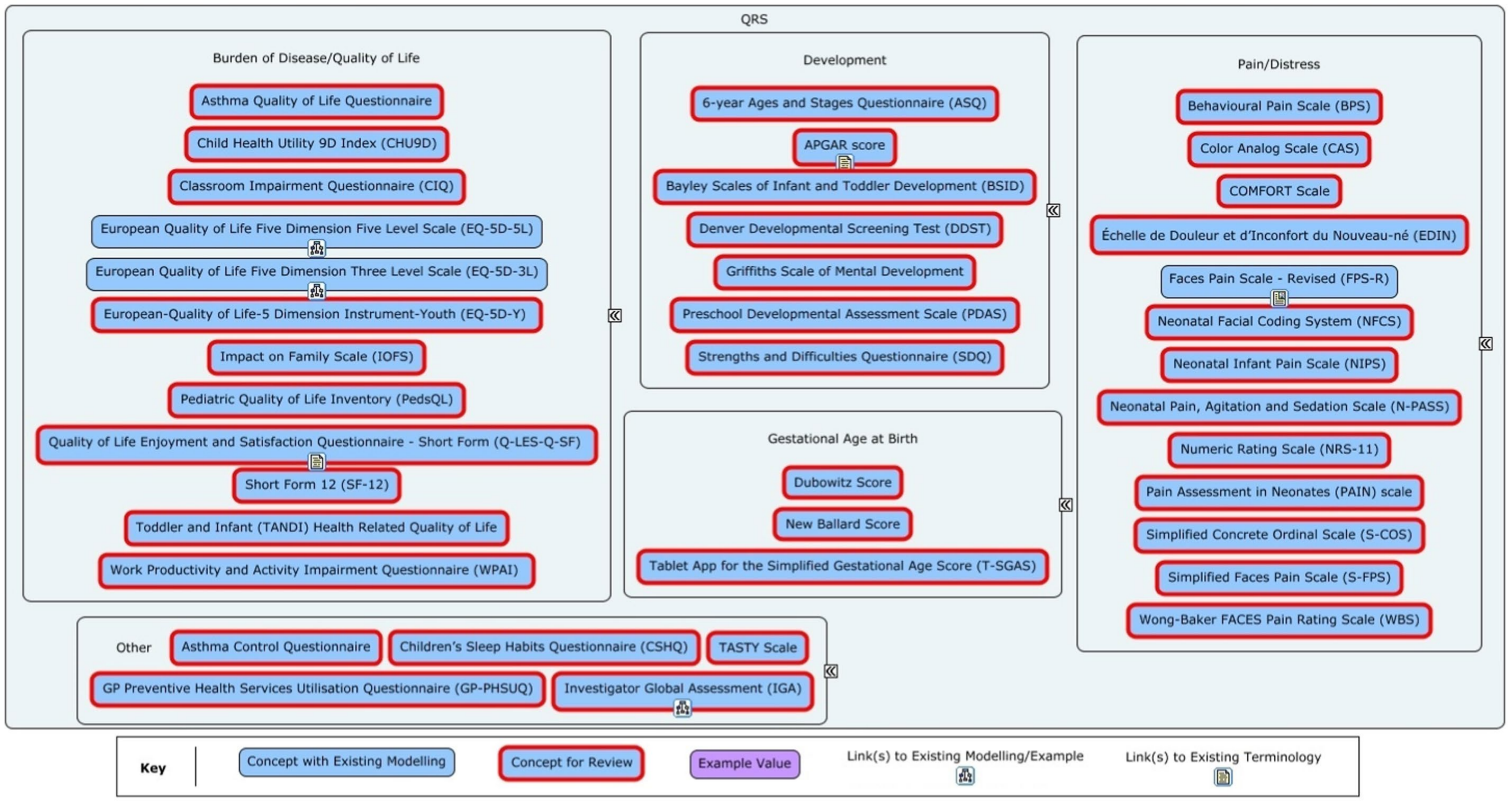
Overview of the Questionnaire, Rating and Scales (QRS) instruments in the initial concept list for the Pediatrics User Guide. The red-outlined concepts marked for review had partial or no modelling available or required a deeper dive to fully understand the data for the concept.

From this list, items that were deemed ineligible (i.e., did not meet the aforementioned criteria) were removed by consensus. Further filtering was required for QRS instruments. First, the same criteria were applied to remove ineligible items. Since most QRS instruments would require new modelling (see Figure 2) and CDISC had limited budget for modelling for QRS instruments, a maximum of 10 QRS concepts could be modelled. Given these constraints, a decision was made to try and identify the most relevant QRS instruments used in paediatric studies, and to prioritize these for modelling. As some QRS instruments are only used for specific paediatric age groups – neonates, toddlers, children, and adolescents – it was decided that the list of QRS instruments identified for modelling should include at least one instrument for each paediatric age group.

Given the level of winnowing required (from 28 to 10 – see results), verbal consensus was not seen as an effective tool. A deterministic ranking-based process was developed for the selection of QRS instruments. Relevant c4c Expert Groups were contacted to take part in the final selection. Members of the groups included clinicians, clinical trials experts, pharmacovigilance experts, etc. There were at least 15 clinicians among the experts, which ensured the clinical relevance of the QRS instruments were adequately considered. Each expert received an Excel spreadsheet (one sheet for each age-group) containing lists of the QRS identified for possible inclusion for each age group. They were asked to identify the most cross-cutting concepts by assigning a rank to them in order of relevance and importance (with a rank 1 indicating the highest relevance/importance). For every QRS instrument on a sheet, the given ranks were averaged over the number of expert responses. The four instruments with the highest average ranking (i.e., those ranked as most relevant/important) were selected from each age group for a total of 10 QRS concepts. This was possible due to the overlap of some QRS instruments across age groups.

### 2.2 Concept modelling

Concept modelling was carried out by CDISC between July and September 2021 as part of the development of CDISC standards. It focused on how a concept is applied and interpreted in a clinical study setting. First, a check was performed to determine whether the concept had been addressed in existing CDISC standards. If not, the concept was required to be sufficiently defined by the concept developers to reach a common understanding with clinical experts. The clinical context was discussed, and the data and metadata needed for fully representing the details of the concept were identified. Concept maps were used to facilitate this process. A concept map is a flowchart that illustrates the steps for recording a concept along with relevant qualifiers. An example of a concept map collected in a clinical study setting is described in the results.

### 2.3 Standards development

Standards development was completed over a six-month period between October 2021 and March 2022. This stage involved the development of metadata and implementation examples to fully represent the concepts in CDISC standards. The CDASH model provided precise and unambiguous language to be used in CRFs to collect the data, and the SDTM model provided a standard way to represent the data in a tabular format. The process involved CDASH and SDTM metadata modelling experts who determined whether new variables or domains were required for new concepts and provided references and links to respective CDASH and SDTM variables for concepts with existing modelling so as not to duplicate content and provide as much information to the users as possible. They developed implementation examples and supporting guidance to help a user understand how to represent the concepts. Metadata developers also had access to a range of CDISC experts from the CDISC Internal Standards Modelling Team, who could provide input and guidance for more complex modelling. A demonstration of an implementation example with CDISC terminology is provided in the results.

### 2.4 Internal review

The draft of the PUG (including all 90 selected concepts, concept maps, their hierarchy with the CDISC domains and implementation examples with metadata) was submitted to the CDISC Global Governance Group (GGG) on 16th March 2022 for approval to be sent for internal review. Approval was received on 25th March 2022. The internal review period started on 29th March 2022 and ran until 19th April 2022. During this time, the PUG was presented to targeted reviewers that included CDISC teams, collaborative groups, and SMEs. The goal of the internal review was to ensure (1) concepts and reuse of previous definitions had been properly represented in the standard, (2) modelling was logically consistent both for the PUG and with the larger CDISC suite of standards, (3) new domains and variables had been aligned properly with existing CDISC standards, and the (4) document met CDISC quality standards.

Comments were managed within the CDISC Jira comment tracking system. Once feedback was received, the CDISC experts categorized the comments with eight disposition codes and then addressed the relevant comments. The disposition codes were defined as follows:

1. Considered for future – The comment will be considered for a future release but not the release associated with this review.
2. Considered, no-action-required – The comment was considered, but nothing is required because of the comment (e.g., a compliment).
3. Not persuasive – The comment is not convincing or is unclear. It is preferable to clarify an unclear comment with the reviewer.
4. Not persuasive with modification – The comment is not convincing, but changes were made based on the content of the comment.
5. Out of scope – The comment is considered not related to the document under review.
6. Persuasive – The change requested in the comment is accepted and will be made as proposed.
7. Persuasive with modification – The comment is accepted, but the change made is different from the proposed solution.
8. Question answered – The comment is a question that has been answered.

### 2.5 Public review

The public review was the next quality step in the CDISC data standards development process. The draft was made available on the CDISC website (registration and login to CDISC wiki required), and any person or organization interested in paediatric research, including experts from industry and academia, patients, caregivers, or parents, could provide comments during the public review. The main purpose of public review was to ensure that neutral, consensus-based data standards were developed and adopted by a diverse global community.

The GGG met on 20th May 2022 for approval of the PUG to be sent for public review. The public review period ran from 6th June 2022 to 25th August 2022. A public review webinar was hosted by CDISC and c4c on 12th July 2022 to introduce the PUG draft to interested members of the public and provide information about how to submit comments. After the public review period, the comments were handled in an identical fashion as the internal review process.

### 2.6 Publication

After the resolution of all comments, the PUG was published on the CDISC website.

## 3 Results

### 3.1 Scoping

The results of the voting on the QRS instruments are shown in Table 1. Initially 28 QRS instruments were identified as being cross-cutting, paediatric-specific, and routinely collected in clinical trials. The selection of 10 QRS instruments for modelling included the top 4 concepts from each age group due to the repetition of some concepts across age groups. The remaining 18 concepts were not taken forward for modelling.

**Table 1:** Average rank for the age-group-specific QRS instruments assigned by the c4c expert team. Cells containing the four best ranks (which indicate the QRS instruments ranked with the highest relevance/importance) for each age group are highlighted in green.

Scale	Average ranking among			
	Neonates	Toddlers	Children	Adolescents
6-Year Ages and Stages Questionnaire (ASQ)		2.67	3.78	
APGAR score	2.75			
Bayley Scales of Infant and Toddler Development (BSID)		1.25		
Child Health Utility 9D Index (CHU9D)			6.33	
Children’s Sleep Habits Questionnaire (CSHQ)			7.67	
Classroom Impairment Questionnaire (CIQ)			8.00	5.33
Color Analog Scale (CAS)			6.83	8.50
COMFORT Scale	2.68	2.67	4.83	3.71
Denver Developmental Screening Test (DDST)	4.25	2.83	2.33	
Dubowitz Score	8.18			
Échelle de Douleur et d’Inconfort du Nouveau-né (EDIN, neonatal pain and discomfort scale)	4.27			
European Quality of Life Five Dimension Instrument-Youth (EQ-5D-Y)			5.00	3.38
Faces Pain Scale-Revised (FPS-R)			5.67	6.33
Griffiths Mental Development Scales (GMDS)		3.89	4.11	
Impact on Family Scale (IOFS)	5.33	3.67	5.83	3.50
Neonatal Facial Coding System (NFCS)	4.25			
Neonatal Infant Pain Scale (NIPS)	5.02			
Neonatal Pain, Agitation and Sedation Scale (N-PASS)	5.1			
New Ballard Score	7.38			
Numeric Rating Scale (NRS-11)			9.83	6.33
Pain Assessment in Neonates (PAIN) scale	6.62			
Pediatric Quality of Life Inventory (PedsQL)			3.50	1.83
Simplified Concrete Ordinal Scale (S-COS)			14	
Simplified Faces Pain Scale (S-FPS)			7.83	
Strengths and Difficulties Questionnaire (SDQ)			7.88	4.25
Tablet App for the Simplified Gestational Age Score (T-SGAS)	6.33			
Toddler and Infant (TANDI) Health-Related Quality of Life		4.83		
Wong-Baker FACES Pain Rating Scale (WBS)			9.83	11

From the long list (Figures 1, 2), 94 concepts were identified for taking forward to the modelling phase. Four further concepts were excluded as follows. Oxygen Saturation could be classified as a vital sign or laboratory test, depending on the method of measurement. This would require complex modelling to fully describe the different scenarios. The budgetary load for this could not be justified based on the relative importance of the concept. In addition, the concept was not considered to be paediatric-specific. Position at birth could have been of interest if it affected outcomes but was not deemed common enough in most paediatric trials to be of interest. Dental examination (assessment of teeth) was not deemed to be commonly performed in clinical trials. Odontogenic status for age was considered a low priority as it would only be used if there was a specific need to assess effects on dental development, and hence it was not considered to be cross-cutting. The remaining 90 concepts moved on to the modelling stage.

### 3.2 Concept modelling

Existing modelling was found to be appropriate for 71 concepts. New modelling was developed for 16 concepts (see Box 1). For three concepts – Visual capacity, Estimated gestational age, and Estimated date of delivery, some modelling already existed, but additional modelling was required. For visual capacity, modelling existed for the ophthalmic examination and Teller Acuity Cards test. New modelling was added for the Cardiff Acuity Test and Sweep Visual Evoked Potential test. For estimated gestational age, new modelling was added to denote the age in days or weeks. Finally, for the estimated date of delivery, terminology existed for representing the date as a reproductive system finding for the mother but not as a characteristic of the child subject in a paediatric study.

Box 1 **List of concepts included in the CDISC Paediatric User Guide.**

Existing modelling sufficient – 71 concepts: Pregnancy conditions, Pregnancy-related event, Pregnancy-related procedure, Perinatal events, Birth complications, Birth trauma, Breast fed indicator, Breast feeding, Nutrition, Musculoskeletal findings, Laboratory tests, eGFR, Pregnancy test, Cardiovascular findings, ECG test results, Male genitalia stage, Male pubic hair stage, Female breast stage, Female pubic hair stage, Testicular volume, Menarche, Date of menarche, Age of menarche, Childbearing potential, Systolic blood pressure, Diastolic blood pressure, Temperature, Respiratory rate, Pulse rate, Heart rate, Mean arterial pressure, Height, Total body Length, Weight, Body mass index (BMI), Body surface area, Head circumference, Birth weight, Mid-upper arm circumference, BMI percentile, Head circumference percentile, Adverse events, Clinical events, Medical history, Congenital malformations, Physical examination, Healthcare encounters, Substance use, Level of education attained, Family history of prespecified condition, Family history of prespecified procedure, Family history of substance use, Family history of prespecified medication, Concomitant medications, Procedures, Exposure, Date of birth, Phenotypic sex, Date of death, Race, Ethnicity, Disposition, Product accountability, Subject identifier, Investigator identifier, Visit number, Visit date, Eligibility criteria, Study arm, VAS representation of disease assessment, Reason not done.

Modelling updated – 3 concepts: Visual capacity, Estimated gestational age, and Estimated date of delivery.

New modelling developed – 16 concepts: Subject status regarding multiple births, Graf classification, Alpha angle, Beta angle, Neurological findings, Hearing capacity, Birth weight status (for gestational age), Birth weight category, Length-for-age z-score, Weight-for-age z-score, Weight-for-length z-score, Head circumference z-score, Level of dehydration, Socio-economic classification, Outcome of study treatment, and Gestational age group at Birth.

**Figure 3:**
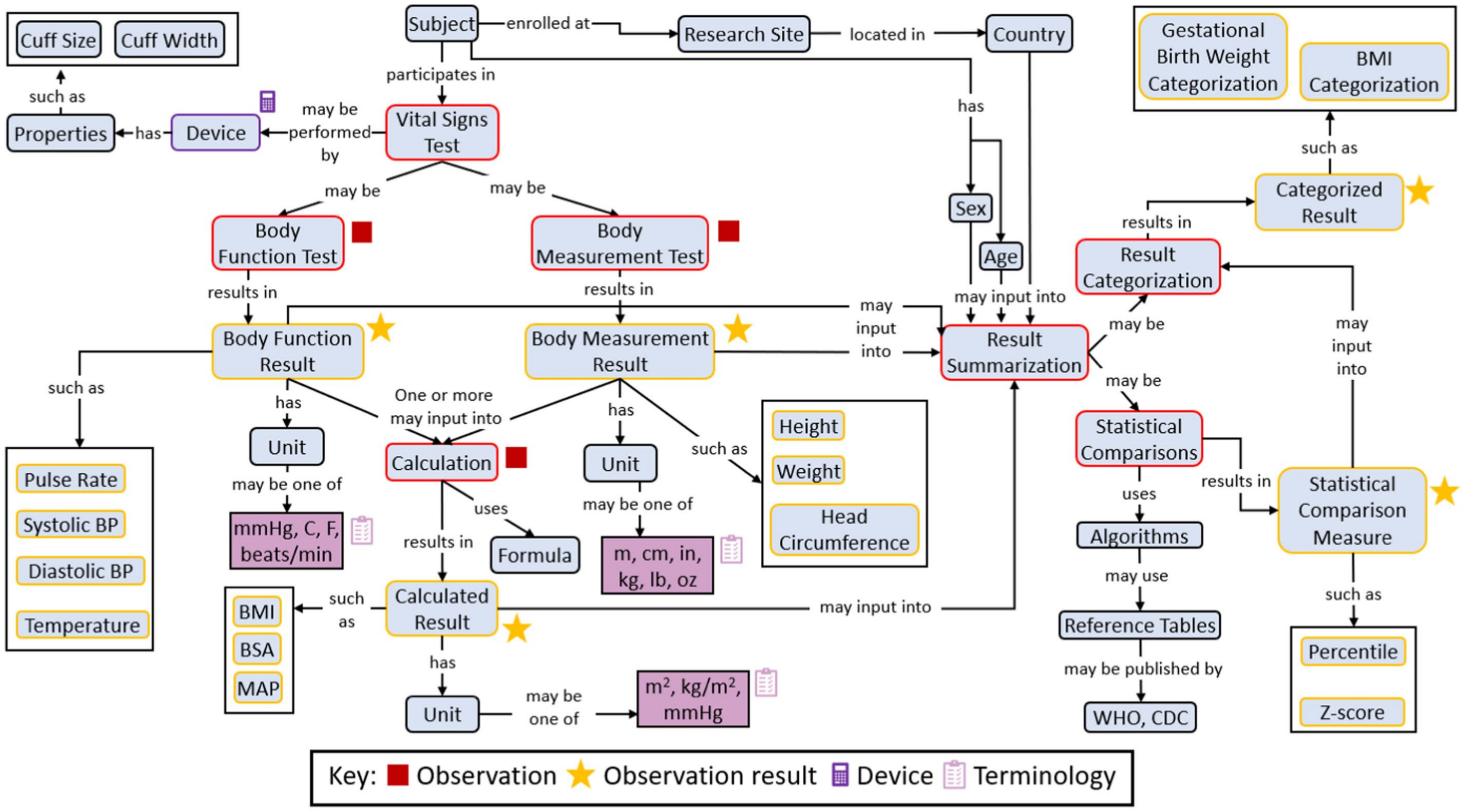
A concept map illustrating “vital signs” concepts as collected for a clinical study.

Figure 3 shows an example concept map illustrating the concept representing the Research Topic of ‘vital signs’ information for a paediatric clinical trial.

### 3.3 Standards development

**Figure 4:**
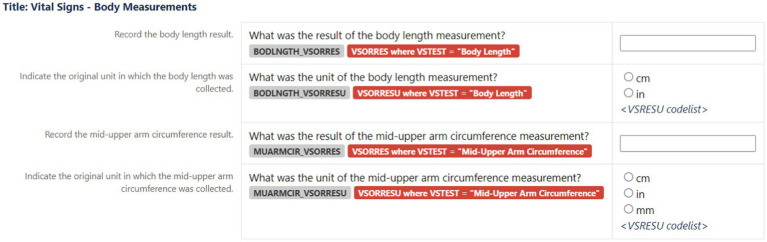
An example to show how CRF metadata (Table 2) transformed into data Research Topic fields for CRFs.

Demonstrations of an implementation example with metadata are shown in Tables 2, 3 and Figure 4. Both the tables are copied verbatim from the PUG while the figure is a screenshot. Table 2 shows the CDASH metadata describing the example CRF in Figure 4, while the Table 3 shows collected data that has been converted to SDTM format, which is a standardized representation of data that is more machine-readable and can be used for downstream purposes such as statistical analysis. In the presented example, a clinical study requires the Research Topic of total body length and mid-upper arm circumference (both of which are concepts). This further requires the Research Topic of two variables for each measurement: one variable to represent the measurement and one variable to represent the unit of that measurement (Figure 4). These four variables are the four rows of Table 2. Column 2 references the CDASH variables corresponding to these collected data items. Column 3 (Question Text) suggests the text for the corresponding question to be asked in the CRF, and column 4 (Prompt) suggests a shorter name for the CRF field, if preferred. Column 5 provides instructions for completing the CRF. Column 6 is the data type for each variable. Column 7 (SDTMIG Target Variables) is the corresponding SDTM variable, while column 8 (SDTMIG target mapping) is the internal CDISC mapping for the conversion. Columns 7 and 8 can both be used to convert CDASH data into SDTM data. Columns 9 and 10 (Controlled Terminology Code List Name and Permissible Values) specify the available units of length.

**Table 2:** Description of the CDASH metadata that is used to generate data Research Topic fields for the CRFs (example shown in Figure 4).

Order	CDASH variable	Question text	Prompt	CRF completion instructions	Type	SDTMIG target variable	SDTMIG target mapping	Controlled terminology code list name	Permissible values	Pre-populated Value	Query display	List style	Hidden
1	BODLNGTH_VSORRES	What was the result of the body length measurement?	Body Length	Record the body length result.	Float	VSORRES	VSORRES where VSTEST = “Body Length”						
2	BODLNGTH_VSORRESU	What was the unit of the body length measurement?	Body Length Unit	Indicate the original unit in which the body length was collected.	Text	VSORRESU	VSORRESU where VSTEST = “Body Length”	(VSRESU)	cm; in				
3	MUARMCIR_VSORRES	What was the result of the mid-upper arm circumference measurement?	Mid Upper-Arm Circumference	Record the mid-upper arm circumference result.	Float	VSORRES	VSORRES where VSTEST = “Mid-Upper Arm Circumference”						
4	MUARMCIR_VSORRESU	What was the unit of the mid-upper arm circumference measurement?	Mid Upper-Arm Circumference unit	Indicate the original unit in which the mid-upper arm circumference was collected.	Text	VSORRESU	VSORRESU where VSTEST = “Mid-Upper Arm Circumference”	(VSRESU)	cm; in				

**Table 3:** Example data for two patients (recorded via CRFs described in Table 2 and Figure 4) as represented in an SDTM table.

Row	Study-ID	Domain	USUBJID	VSSEQ	VSTESTCD	VSTEST	VSORRES	VSORRESU	VSSTRESC	VSSTRESN	VSSTRESU	VSLOBXFL	VISTNUM	VISIT	VSDTC
1	PED-011	VS	PED-011-001	1	BODLNGTH	Body Length	62.0	cm	62.0	62.0	cm	Y	1	Baseline	2021-06-19
2	PED-011	VS	PED-011-001	2	MUARMCIR	Mid-Upper Arm Circumference	149	mm	14.9	14.9	cm	Y	1	Baseline	2021-06-19
3	PED-011	VS	PED-011-002	1	BODLNGTH	Body Length	25.4	in	64.5	64.5	cm	Y	1	Baseline	2021-07-21
4	PED-011	VS	PED-011-002	2	MUARMCIR	Mid-Upper Arm Circumference	5.9	in	15.0	15.0	cm	Y	1	Baseline	2021-07-21

Table 3 shows the data represented according to the SDTMIG specification for the representation of vital signs information, which includes standard variable names and their corresponding definitions (shown in parentheses in the following description of Table 3). The table shows the 2 concepts in the VSTEST (Vital Signs Test Short Name) column that were collected for the 2 subjects identified in the USUBJID (Unique Subject Identifier) column in the clinical trial identified in the STUDYID (Study Identifier) column. The DOMAIN (Domain Abbreviation) column denotes that both measurements fall under the Vital Signs domain in CDISC. The VSSEQ (Sequence Number) column notes a unique sequence number of measurements within a subject and is used for ordering data within a dataset. The VSORRES (Result or Finding in Original Units) and VSORRESU (Original Units) columns denote the collected measurements and units, respectively. Since the study sponsor required measurements in centimetres, the VSSTRESC (Character Result/Finding in Standard Units), VSSTRESN (Numeric Result/Finding in Standard Units), and VSSTRESU (Standard Units) columns store the measurement transformed into standardized units (in both character and numeric formats) and units, respectively. The last four columns all relate to timing information. The VSLOBXFL (Last Observation Before Exposure Flag) column indicates whether this was the last measurement before exposure to the study intervention, the VISITNUM (Visit Number) column shows the visit number for the patient, the VISIT (Visit Name) column shows name of the visit in terms of the trial, and the VSDTC (Date/Time of Measurements) column shows the visit date.

### 3.4 Internal review

Further analysis of the internal review was not possible as the process is internal to CDISC, and individual comments are not shared outside of the CDISC development team. The summary of the comments and CDISC dispositions is shown in Table 4.

**Table 4:** Summary of the categorization of internal review comments.

Components	CDISC disposition								
	Considered for future	Considered, no action required	Not persuasive	Not persuasive with modification	Out of scope	Persuasive	Persuasive with modification	Question answered	Total
Body System Assessments					1	13	1	1	16
Demographic Information		1	1	1		6	1	2	12
Diet and Nutrition						5	1	1	7
General				1		7			8
Information about family	1	1	1			7			10
Laboratory Assessments						1	1	1	3
Medical Conditions			2	1		2	1	3	9
Pregnancy and Birth				1		3	1	1	6
Study Conduct	1			1		5			7
Treatment						1			1
Vital Signs				1		11	1		13
Total Unique Issues	2	2	4	6	1	61	7	9	92

### 3.5 Public review

A total of 45 comments were received during the public review period. The comments were relatively minor and did not cause significant delay. As necessary and/or required, they were discussed and resolved with the CDISC Internal Standards Modelling Team or the Controlled Terminology team. A further 17 comments were received from the Food and Drug Administration (FDA) after the public review period ended. These comments were discussed with the FDA PUG reviewer, and minor updates were made to the PUG, which did not require additional input from the Paediatric SMEs. A summary of the CDISC disposition of the 62 comments is shown in Table 5. None of the comments had the disposition ‘Considered for future’. Examples of public review comments and dispositions are shown in Table 6.

**Table 5:** Summary of the categorizations of the public review comments.

Components	CDISC disposition							
	Considered, no action required	Not persuasive	Not persuasive with modification	Out of scope	Persuasive	Persuasive with modification	Question answered	Total
Body System Assessments			1		10	2	6	19
Demographic Information	1	1			4	3	1	10
Diet and Nutrition						1	2	3
General					2			2
Information about family					1	1		2
Laboratory Assessments					1			1
Medical Conditions				1	2	1	2	6
Pregnancy and Birth	1			1	2	2	2	8
Questionnaires Ratings Scales					1		3	4
Reproductive Development					1			1
Study Conduct						1		1
Treatment					1			1
Vital Signs	1		1		2			4
Total Unique Issues	3	1	2	2	27	11	16	62

**Table 6:** Examples of public review comments related to recording of age.

Issue key	Summary	Description	Status	CDISC disposition	CDISC disposition description
PEDIAC-101	LAR and adolescent reaching age to consent	Terminology not yet available. The concept is acceptable; just need the terminology.	Resolved	Persuasive with modification	The first sentence was updated from “obtained from a legally acceptable representative of the subject, such as the subject’s parent(s) or legal guardian/custodian” to “obtained from the subject’s parent(s), legal guardian/custodian, or other legally authorized representative (LAR).” The example was also updated to include representations of the withdrawal of consent and assent.
PEDIAC-117	Age	The age “at the time of the test” would be used to determine appropriate normal ranges, but I do not see any discussion about re-collecting the age at the time of the measurements. It was mentioned at a CDASH all-hands meeting that some clinical sites enter the age of the subject at each visit, since the “normal” values can change as the child’s age changes, which affects the meaning of the results (normal/abnormal high/low). Would you suggest a way to collect age at each visit (particularly when collecting BRTHDAT/BRTHDAT is not allowed due to privacy)?	Resolved	Not persuasive with modification	If a full date of birth (DOB) has not been collected for privacy reasons, it’s likely that the same privacy reasons would mean that age should not be collected and represented at regular time points either, because doing so would allow the possible full value of the DOB to be narrowed down. This is especially true in pediatric studies where age would have to be collected frequently to allow the correct reference ranges to be applied, given that pediatric reference ranges may vary frequently according to age. The “Date of Birth and Age” section was updated to include a recommendation that, in this situation, sponsors should use an alternative approach. Both the “Laboratory Assessments” and “Body Function Tests” sections were updated to include additional information about normal/reference ranges, with reference to the updated “Date of Birth and Age” section regarding the application of age-related ranges.
PEDIAC-151	Subject or the subject’s age	Suggest adding the CDASH recommendation that, if collecting age, be sure to collect the date that the age was collected; this allows for the calculation of age at other time points.	Resolved	Persuasive	The text indicating the importance of collecting (or deriving) date of Research Topic with age was moved from Example 2 into the main narrative. The text was also modified to indicate that collecting date of Research Topic with age is a CDASH recommendation.
PEDIAC-99	Age groups and definitions	Would it be possible to define terms such as neonate, infant, child, adolescent in this TAUG? I beleive that difference regulators may have slightly different age ranges, but it would be good if the terms could have a general definition.	Resolved	Persuasive	NCI definitions were added to the Glossary and Abbreviations page for “Fetus,” “Newborn/Neonate,” “Infant,” “Child” and “Adolescent.”

The CDISC dispositions and resolution status are shown.

While a review of all 62 comments is not feasible to report in this paper, two major themes emerged – (1) recording of age and (2) differentiation between the mother and the baby during pregnancy. Four comments concerned if, when and in what format age should be recorded without narrowing down the date of birth (which is considered protected information), informed consent and definitions of the age-groups used for the QRS selections. The resolution of three of these comments was persuasive (one with modifications). One comment was not persuasive with modification. This was regarding the recording of detailed age at the time of a test, as normal ranges for the tests are often age dependent. While this was not persuasive due to the narrowing down of the date of birth, text was added to make users aware of such situations, and to include a reference to an article discussing alternative approaches that ensuring subject privacy while maintaining data utility. The comments, along with dispositions and resolution status, are shown in Table 6 (copied verbatim – including capitalisations, typos, and American English spellings, from the Public Review document available on the CDISC website). Five comments concerned separating the child and mother’s data while keeping a link intact. Three of the comments were persuasive (two with modification), one was a question that was answered, and one was out of scope.

The complete public review spreadsheet with all comments can be viewed on the PUG publication link provided below.

### 3.6 Publication

The PUG was initially presented to the CDISC GGG on 11th November 2022 for approval for publication. It was submitted a second time to the GGG on 3rd February 2023 after GGG and FDA comments were resolved. The PUG was published on the CDISC website on 22nd February 2023. It can be viewed and downloaded at https://www.cdisc.org/standards/therapeutic-areas/pediatrics/pediatrics-user-guide-v1-0. A CDISC login (different from the CDISC wiki login used for public review) is required, and registration is free.

## 4 Discussion

The PUG provides clear guidelines on how to collect and record paediatric cross-cutting data items. Long-term usage of these guidelines will result in more standardized paediatric data, enabling potential pooling of data across multiple trials. In a field like paediatrics, where disease populations are small, pooled data have multiple applications, including serving as a comparator arm, safety data analyses, larger post-hoc analyses with enhanced statistical power, analysis of under-represented subgroups and pragmatic clinical trials (26–29). Such applications are not restricted to paediatrics and can be extended to other fields with smaller populations, such as the rare disease field. While not all paediatric diseases are rare, most rare diseases affect the paediatric population, and there are important overlaps between the two fields (30–32). CDISC has developed a separate Rare Disease TAUG for standardization of rare disease terminology that was released in late 2023 (33).

The added value of CDISC developing the PUG with c4c was the access to 21 countries and about 240 sites which improved the quality of selection as well as the implementation facilitation. The National Hubs of each country were able to provide nationally relevant information during the scoping process. While the exact role of each National Hub was not tracked, as an example the Belgian National Hub - Belgium Paediatric Clinical Research Network (BPCRN) located at Ghent University played an important role in the scoping and provided expert opinion. A paediatric resident, a data manager and a clinical research coordinator were involved in the review of 50+ clinical trials performed between 2018 and 2020. Thirty-five cross-cutting terms were identified and shared with experts from 15 sites within Belgium. Based on expert feedback with a combined paediatric experience of 50+ years five additional cross-cutting terms were added to the list.

As a part of academic outreach, c4c has been working on introducing CDISC standards to the European Reference Networks (ERNs) (34). There are 24 ERNs across Europe that aim to tackle complex rare diseases by establishing registries with patient-level data. Each ERN tackles a disease or different therapeutic area (e.g., metabolic disease, paediatric cancers, kidney disease). Each ERN also develops its data dictionary, though their sizes vary greatly, containing between 16 and over a thousand data elements. While the PUG itself may have limited utility for ERNs due to its cross-cutting nature, in general, CDISC standards would benefit the academic institutions involved with the ERNs. As of October 2023, MetabERN (an ERN addressing metabolic disease) is conducting desk research to evaluate the benefits of CDISC standards in paediatrics for ERNs. Tasks have included translating case report forms to CDISC standards and development of the implementation of the CDISC PUG for ERNs.

The implementation of PUG remains a challenge. While c4c collaborated with CDISC in the development, it has no say in whether institutions choose to use it for their clinical trials. Institutions that are not using CDISC standards or do not know about CDISC standards may be hesitant in switching to a completely new standards – particularly, in the absence of short-term benefits. With academic partners heavily involved in the development of the PUG, several academic colleagues gained first-hand experience in the development of CDISC standards and how they are presented for use. The PUG was widely promoted within these institutions. Concurrently, c4c has promoted the PUG across all 20 National Hubs (comprising about 240 sites) and presented it at various conferences and symposia. It is hoped that this promotion strategy will lead to continued adoption of CDISC standards across institutions, national networks, and nations. In the future, the PUG will be expanded both in terms of terminology as well as utility (e.g., guidance for statistical analysis) while continuing to be freely available on the CDISC website. Widespread adoption along with expansion will enable the applications of pooled data mentioned above.

While the CDISC PUG is a resource for clinical trials, similar methodologies (concept modelling, human and machine-readable formats, and implementation examples) can be used in the standardization of real-world data (RWD). Two common formats for RWD data are the Observational Medical Outcomes Partnership Common Data Model (OMOP-CDM) and Fast Healthcare Interoperability Resources (FHIR). Having direct mappings from FHIR and OMOP to CDISC standards could enable the use of RWD for clinical studies. FHIR, OMOP and CDISC have heavily collaborated to develop these mappings. A FHIR to CDISC joint-mapping was released in 2021 (35). A similar mapping from OMOP to CDISC was presented in 2022 (36). Reverse mappings from RWD to CDISC have also been developed (37, 38). c4c continues to engage with all three standards and foster collaboration (39). While these mappings can provide pathways for the standardization of RWD, it must be noted that some loss of information is associated with all such mappings.

### 4.1 Limitations

As with any consensus-based methodology, the final outcome is dependent on subjective judgements. A different set of experts may have arrived at a slightly different set of terms. While every effort was made to achieve unanimous consensus, this wasn’t always possible. The large group of experts involved in the scoping process substantially increased the timelines. The balance between greater involvement and timelines is a challenge. As this was a first effort towards paediatric data standardization, greater involvement was deemed crucial. Additionally, budgetary constraints necessitated certain cost-cutting decisions. As mentioned, only ten QRS instruments could be modelled. Moreover, unlike many other CDISC user guides and TAUGs, the PUG did not include the Analysis Data Model (ADaM) standards. ADaM is a CDISC foundational standard that defines data and metadata standards for the generation, replication, and review of clinical trial statistical analyses (40, 41). SDTM tables serve as inputs for ADaM. A future version of the PUG will likely include ADaM standards. Future versions are expected to be developed within expedited timelines since the current version has set a solid base for the methodologies involved.

## 5 Conclusion

We developed a Pediatrics User Guide consisting of cross-cutting concepts routinely collected in paediatric clinical trials through a rigorous consensus-based process. We described in detail the stages behind the development of CDISC user guides that can aid sponsors of paediatric clinical trials, including academic institutions, understand CDISC standards better. Continued use of the PUG has the potential to enable interoperability across paediatric clinical trial datasets.

## Author’s note

The word ‘paediatric’ is spelled in British English throughout the manuscript except in names of materials developed in the United States that use American English spellings (e.g., Pediatrics User Guide, public reviews comments, PedsQL).

## Data availability statement

The datasets presented in this study can be found in online repositories. The names of the repository/repositories and accession number(s) can be found in the article/supplementary material.

## Author contributions

JO: Data curation, Formal analysis, Investigation, Methodology, Resources, Supervision, Visualization, Writing – original draft, Writing – review & editing, Conceptualization. AS: Writing – review & editing, Writing – original draft, Visualization, Validation, Resources, Methodology, Investigation, Formal analysis, Conceptualization. BA: Validation, Data curation, Writing – review & editing, Writing – original draft, Supervision, Resources, Methodology, Investigation, Formal analysis, Conceptualization. CE: Data curation, Writing – review & editing, Writing – original draft, Supervision, Resources, Methodology, Investigation, Formal analysis, Conceptualization. GC: Writing – review & editing, Writing – original draft, Resources, Methodology, Investigation, Formal analysis, Data curation, Conceptualization. ED: Conceptualization, Writing – review & editing, Writing – original draft, Resources, Methodology, Investigation, Formal analysis, Data curation. DK: Data curation, Writing – review & editing, Writing – original draft, Supervision, Resources, Investigation, Formal analysis, Conceptualization. RC: Conceptualization, Formal analysis, Investigation, Methodology, Resources, Supervision, Writing – original draft, Writing – review & editing, Data curation. MW: Conceptualization, Formal analysis, Investigation, Methodology, Resources, Writing – original draft, Writing – review & editing, Data curation. TB: Conceptualization, Data curation, Formal analysis, Investigation, Methodology, Resources, Writing – original draft, Writing – review & editing. AP: Writing – review & editing, Writing – original draft, Resources, Project administration, Methodology, Investigation, Formal analysis, Data curation, Conceptualization. FM: Data curation, Formal analysis, Administration, Writing - first draft, Writing – review & editing. SM: Data curation, Formal analysis, Investigation, Methodology, Resources, Writing – original draft, Writing – review & editing. LP: Writing – review & editing, Writing – original draft, Resources, Methodology, Investigation, Formal analysis, Data curation. CA: Writing – review & editing, Writing – original draft, Resources, Methodology, Investigation, Formal analysis, Data curation. JT: Data curation, Formal analysis, Investigation, Methodology, Resources, Writing – original draft, Writing – review & editing. SW: Data curation, Formal analysis, Investigation, Methodology, Resources, Writing – original draft, Writing – review & editing. SN: Conceptualization, Data curation, Formal analysis, Investigation, Methodology, Resources, Supervision, Writing – original draft, Writing – review & editing. RL: Conceptualization, Data curation, Formal analysis, Investigation, Methodology, Resources, Supervision, Writing – original draft, Writing – review & editing, Funding acquisition, Project administration. RM: Conceptualization, Data curation, Formal analysis, Investigation, Methodology, Resources, Supervision, Writing – original draft, Writing – review & editing, Visualization. VS: Conceptualization, Data curation, Formal analysis, Funding acquisition, Investigation, Methodology, Resources, Supervision, Writing – original draft, Writing – review & editing.
